# Genetic Connectedness Between Norwegian White Sheep and New Zealand Composite Sheep Populations With Similar Development History

**DOI:** 10.3389/fgene.2020.00371

**Published:** 2020-04-24

**Authors:** Hinayah Rojas Oliveira, John Colin McEwan, Jette Jakobsen, Thor Blichfeldt, Theodorus Meuwissen, Natalie Pickering, Shannon Marie Clarke, Luiz F. Brito

**Affiliations:** ^1^Department of Animal Sciences, Purdue University, West Lafayette, IN, United States; ^2^Centre for Genetic Improvement of Livestock, Department of Animal Biosciences, University of Guelph, Guelph, ON, Canada; ^3^AgResearch Limited, Invermay Agricultural Centre, Mosgiel, New Zealand; ^4^The Norwegian Association of Sheep and Goat Breeders, Ås, Norway; ^5^Department of Animal and Aquacultural Sciences, Norwegian University of Life Sciences, Ås, Norway; ^6^Focus Genetics, Napier, New Zealand

**Keywords:** admixture, gametic phase, homozygosity, inbreeding, linkage disequilibrium

## Abstract

The Norwegian White sheep (NWS) and New Zealand Terminal Sire Composite (NZC) sheep breeds have been developed based on crossing of multiple breeds, mainly of Northern European origin. A close genetic relationship between these populations could enable across-country genomic evaluations. The main objectives of this study were to assess the genetic connectedness between Norwegian and New Zealand sheep populations and estimate numerous genetic diversity metrics for these two populations. A total of 792 NWS and 16,912 NZC animals were genotyped using a high-density Illumina SNP chip panel (∼606K SNPs). The NZC animals were grouped based on their breed composition as: Finn, Lamb Supreme, Primera, Texel, “Other Dual Purpose”, and “Other Terminal Sire”. The average level of linkage disequilibrium ranged from 0.156 (for Primera) to 0.231 (for Finn). The lowest consistency of gametic phase was estimated between NWS and Finn (0.397), and between NWS and Texel (0.443), respectively. Similar consistency of gametic phase was estimated between NWS and the other NZC populations (∼ 0.52). For all composite sheep populations analyzed in this study, the majority of runs of homozygosity (ROH) segments identified had short length (<2,500 kb), indicating ancient (instead of recent) inbreeding. The variation in the number of ROH segments observed in the NWS was similar to the variation observed in Primera and Lamb Supreme. There was no clear discrimination between NWS and NZC based on the first few principal components. In addition, based on admixture analyses, there seems to be a significant overlap of the ancestral populations that contributed to the development of both NWS and NZC. There were no evident signatures of selection in these populations, which might be due to recent crossbreeding. In conclusion, the NWS composite breed was shown to be moderately related to NZC populations, especially Primera and Lamb Supreme. The findings reported here indicate a promising opportunity for collaborative genomic analyses involving NWS and NZC sheep populations.

## Introduction

The Norwegian White Sheep (NWS) is a composite breed that accounts for 70–75% of the total Norwegian sheep population. This breed is well known for its prolificacy and high growth rates. Sheep in Norway originates from the Northern European short tail breeds (Drabløs, 1997). In the 18th and 19th centuries, better-performing breeds (e.g., Merino for wool production; Oxford Down, Shropshire, Southdown, Leicester, Cheviot, Blackface, and Southerland for meat production) were imported from the United Kingdom and other European countries and used for crossing with Norwegian breeds. Subsequently, three distinct breeds Dala, Rygja and Steigar highly influenced by the imports were formed in the first half of the 20th century. Crossing between these three breeds along with imported Texel and Finn sheep took place in the second half of the 20th century. The composite NWS was officially formed in 2000 including all aforementioned breeds. Nowadays, the NWS is considered a dual-purpose breed (meat and wool), with large emphasis on meat production traits. The NWS breeding program is well organized and has resulted in substantial annual genetic progress for growth and carcass yield, reduced subcutaneous fat, and increased litter size and milking ability (NSG, 2019).

The development history of the New Zealand sheep breeds is somewhat similar to the NWS, with regards to the founder breeders used in their formation and crossbreeding schemes (Brito et al., 2017a). Considering the high genetic variability in each of these composite populations, a collaborative initiative could be a feasible alternative to increase the accuracy of genomic breeding values and other genomic analyses. Benefits may be two-fold, firstly to enlarge the training population of each country and secondly to predict breeding values for traits recorded in a single population (e.g., meat quality, methane emissions).

The genetic connectedness between these two populations can be determined based on the consistency of gametic phase (assessed based on linkage disequilibrium – LD between single nucleotide polymorphisms – SNPs and quantitative trait loci – QTL), as well as other genetic diversity metrics, including admixture and population structure (Brito et al., 2017a; Prieur et al., 2017). Therefore, combining animals from breeds with similar development history can be an option to overcome the small size of training population for certain traits in each population, especially if the divergence between breeds is recent (Gautier et al., 2007; de Roos et al., 2008). The New Zealand sheep industry is characterized by a high proportion of composite breeds and crossbreed animals (Blair, 2011; Brito et al., 2017a), with various overlapping founder breeds in comparison to the NWS. However, the genetic similarity between NWS and NZC sheep populations has not yet been investigated. Knowledge on the genetic diversity and connectedness between NWS and NZC populations will contribute to a better understanding of the development history of both populations and might result in important practical applications. Thus, the main objectives of this study were to: (1) assess the genetic diversity of NWS and NZC sheep populations based on various metrics; and (2) estimate the genomic connectedness between both populations.

## Materials and Methods

All data used in this study were obtained from existing databases made available by the Norwegian Association of Sheep and Goat Breeders (NSG; Ås, Norway) and Animal Genomics (AgResearch; Mosgiel, New Zealand). Therefore, no Animal Care Committee approval was necessary for the purposes of this study.

### Genotypic Data and Quality Control

A total of 792 NWS and 16,912 NZC animals were genotyped using a high-density (HD) SNP panel (Ovine Infinium^®^ HD SNP Beadchip; Kijas et al., 2014). The NZC animals were grouped based on their recorded breed composition as: Finn, Lamb Supreme, Primera, Texel, “Other Dual Purpose”, and “Other Terminal Sire”. The NZC breed groups were formed following Brito et al. (2017a). Note that both Finn and Texel were derived from sampling flocks derived from animals imported to New Zealand in the late 1980s and as such would have a strong population bottleneck. In order to avoid bias due to small sample size (Brito et al., 2017b), only populations that had at least 50 genotyped animals were included in this study. The threshold of 50 animals was defined based on preliminary analysis. In addition, similar thresholds were used in other genetic diversity studies, e.g., Kijas et al. (2012), Prieur et al. (2017), and Brito et al. (2017b).

The genotypic quality control was performed using the PLINK 1.9 software (Purcell et al., 2007), separately for each population, and considering all sheep populations together (specification of the quality control used for the calculation of each diversity metric are described later on). In brief, SNPs with unknown or duplicated genomic positions and/or located in the sexual chromosomes, minor allele frequency (MAF) lower than 0.01, call rate lower than 95%, and extreme departure from the Hardy Weinberg equilibrium (*p*-value < 10^–15^) were excluded. The number of genotyped animals in each population, based on birth year, and the descriptive statistics of the quality control are shown in Table 1.

**TABLE 1:** Descriptive statistics of the genomic datasets used for the analyses.

**Country**	**Breed**	**Number of animals**	**Year of birth**	**Number of SNPs remaining**	**Number of SNPs removed**		
					^5^**MAF**	^6^**CR**	^7^**HWE**
Norway	^1^NWS	792	1977 to 2016	526,044	34,189	16,141	793
New Zealand	Finn	50	1997 to 2005	482,501	79,316	15,350	0
	Primera	8,554	2008 to 2016	502,238	26,145	14,096	34,688
	Texel	220	1985 to 2016	504,836	57,631	14,551	149
	^2^DP	1,831	1996 to 2016	526,874	30,492	17,401	2,400
	^3^LambSup	6,092	1995 to 2016	525,952	29,300	13,626	8,289
	^4^TS	165	2005 to 2016	526,175	34,048	16,893	51
**All**		**17,704**	**1977 to 2016**	**523,355**	**25,031**	**13,880**	**14,901**

*^1^NWS, Norwegian White Sheep; ^2^DP, “Other Dual Purpose”; ^3^LambSup, Lamb Supreme; ^4^TS, “Other Terminal Sire”, ^5^MAF, minor allele frequency lower than 0.01; ^6^CR, call rate lower than 95%; ^7^HWE, Hardy Weinberg equilibrium (*p-value* < 10^–15^).*

### Population Characterization and Genetic Diversity Metrics

#### Linkage Disequilibrium

The extent of linkage disequilibrium (LD) was calculated for each breed group using the –r2 flag available in the PLINK 1.9 software (Purcell et al., 2007). Therefore, LD was calculated as the squared correlation between two alleles at different loci (Hill and Robertson, 1968), i.e.,:

$$\mathrm{LD}=\frac{D^{2}}{f\,\left(A\right)\,f\,\left(a\right)\,f\,\left(B\right)\,f\,\left(b\right)},$$

where D = f(AB) − f(A)f(B), and f(AB), f(A), f(a), f(B) and f(b) are observed frequencies of AB, A, a, B, and b, respectively. Within-population quality control was used to calculate LD for each breed (Table 1).

Average LD values were obtained through a binning approach, in which SNP pairs were sorted into one of 20 bins, based on pair-wise marker distances. The 20 distance bins (described later) were defined to represent the LD decay, as suggested by Barbato et al. (2015). Thus, as defined in preliminary analysis (results not shown), bins reported in this study were required to have at least 50 pairwise estimates and were defined as: lower than 0.01 Mb, from 0.01 until 0.10 defined every 0.01 Mb, from 0.1 to 1 Mb defined every 0.10 Mb, and greater than 1.10 Mb.

#### Consistency of Gametic Phase

Consistency of gametic phase was determined by calculating the square root of the LD values and adding the sign obtained from the disequilibrium (D) metric, as used in the calculation of LD. The *D* values were calculated using the *–dprime-signed* option available in the PLINK 1.9 software (Purcell et al., 2007). Thereafter, the consistency of gametic phase was assumed as the Pearson correlation coefficient between each two breed-group pair, using the signed-squared-root values. The breakdown in the consistency of gametic phase across distances was determined based on the same bins described above. Only SNPs in common (after within-population quality control) among all populations were used to calculate consistency of gametic phase.

#### Proportion of Polymorphic SNPs and Distribution of SNPs by MAF Range

The proportion of polymorphic SNPs (after within-population quality control) for each population was calculated based on SNPs with MAF greater than 0.01 (1%). The distribution of SNPs was calculated for 10 MAF range bins: from 0.01 until 0.50 defined every 0.05 points in MAF.

#### Heterozygosity

The observed heterozygosity (H_O_) per animal, within population, was calculated as the total number of heterozygotes divided by the total number of genotypes. The H_O_ was compared to the expected heterozygosity (H_E_) under Hardy-Weinberg Equilibrium. These estimates were calculated after performing the genotypic quality control for each population (Table 1), except the Hardy Weinberg equilibrium criteria. Both metrics were calculated using the *–hardy* option in PLINK 1.9 (Purcell et al., 2007).

#### Average Pairwise Genetic Distance

The average pairwise genetic distance between individuals from each population was calculated as one minus the average proportion of alleles shared between two individuals (D_ST_). Thus, the D_ST_ was calculated using the *–genomic* option available in the PLINK 1.9 software (Purcell et al., 2007) as:

$${\text{D}}_{\text{ST}}=\frac{\mathrm{IBS2}+\left(0.5\times \mathrm{IBS1}\right)}{\text{m}},$$

where IBS1 and IBS2 are the number of loci that share 1 or 2 alleles identical-by-state (IBS), respectively, and m is the total number of loci. LD pruning was performed prior the calculation of the genetic distance, by using the *–indep* option of PLINK 1.9, considering a window size of 50 SNPs, 5 SNPs to shift the window at the end of each step, and the variance inflation factor equal to 2 (PLINK default parameter). A genotypic quality control considering all sheep populations together was used to estimate the average pairwise genetic distance.

### Runs of Homozygosity (ROH)

Runs of homozygosity were identified using the *–homozyg* option available in the PLINK 1.9 software (Purcell et al., 2007), considering the default options. The default options included the use of scanning window containing 50 SNPs and at most 1 heterozygous call in a ROH. In addition, the maximum average distance between SNPs in each ROH was set as 50 kb and the maximum distance allowed between consecutive SNPs in the same ROH was 1,000 kb. The minimum number of SNPs to be considered a ROH was calculated following Lencz et al. (2007), in order to minimize the probability of homozygous sequences to be observed by chance. The percentage of false positive ROH was set to 5% (i.e., *p*-value < 0.05).

#### Inbreeding Coefficients

Three different measurements of genomic inbreeding were calculated for all sheep populations: (1) genomic inbreeding based on excess of homozygosity; (2) genomic inbreeding based on the variance of additive genotypes; and (3) ROH-based inbreeding. The genomic inbreeding based on excess of homozygosity was calculated as currently performed in PLINK 1.9 (Purcell et al., 2007), using all genotyped animals and SNPs that remained from the genotypic quality control performed individually for each population (Table 1). The genomic inbreeding based on the variance of additive genotypes was calculated as the diagonal of the genomic relationship matrix (**G**, calculated as in VanRaden, 2008, method 1, considering the observed allele frequencies) minus 1. ROH-based inbreeding was calculated as the genome length covered by ROH divided by the total genome length across all 26 autosomes. Pedigree-based inbreeding was also calculated for the NWS animals, using the Meuwissen and Luo (1992) algorithm, as implemented in the INBUPGF90 software (Misztal et al., 2002). All animals related to the genotyped animals (i.e., that had any relationship with genotyped animals) were included in the analyses (*n* = 27,114 animals).

### Clustering Populations and Admixture Analysis

#### Principal Component Analysis (PCA)

Principal component analysis was performed to investigate the genomic similarities between NWS and NZC sheep populations, using the –pca flag available in the PLINK 1.9 software (Purcell et al., 2007). Principal components were estimated based on the variance-standardized genomic relationship matrix (**G**, calculated as in VanRaden, 2008, method 2), in which the covariance for each SNP was divided by the respective SNP’s variance (calculated from the observed MAF). LD pruning was also performed and the genotypic quality control was performed considering all populations together.

#### Admixture Analysis

The genomic make-up (population structure) of each animal was assessed using the ADMIXTURE software (Alexander et al., 2009). In summary, this software clusters individuals into k pre-defined ancestral groups based on distinctive allele frequencies. The optimal *k* value was defined through a 10-fold cross-validation procedure, with k ranging from 1 to 25. Thus, the *k* value with the lowest cross-validation error was assumed as the optimal *k* value to represent the optimal number of ancestral breeds. Standard errors were estimated using 100 bootstrapping replicates, and the convergence acceleration method used was the quasi-Newton method, with *q* = 3 secant conditions (Alexander et al., 2009).

The genomic dataset after performing quality control considering all populations together and linkage disequilibrium pruning was used. As sample size can affect the Admixture analysis, a randomly selected sample of 150 animals from each sheep population was used for the analyses.

#### Genomic Population Tree

The genomic population tree was created using the IBS matrix generated by the *–matrix* option in PLINK 1.9 (Purcell et al., 2007). An average distance matrix among populations was calculated as 1 – (average IBS), which was used to plot the genomic population tree using the *plot(hclust)* function available in R (R Core Team, 2013). The same dataset described for the admixture analysis was used to create the genomic population tree.

### Signatures of Selection

#### F_ST_ Statistic

F_ST_ was calculated for each SNP as the squared deviation of the average frequency in the NWS population from the average frequency across NZC populations (i.e., pairwise comparisons) divided by the allele frequency variance. This was implemented using the *–fst* option available in PLINK 1.9 (Purcell et al., 2007). Only SNPs that were in common for all breed groups were used to estimate the F_ST_ statistic. Genotypic quality control was performed considering all populations together. LD pruning was also performed. In this context, SNPs with F_ST_ values greater than the average plus three standard deviations from the mean were considered to be under selection.

## Results

### Population Characterization and Genetic Diversity Metrics

**FIGURE 1:**
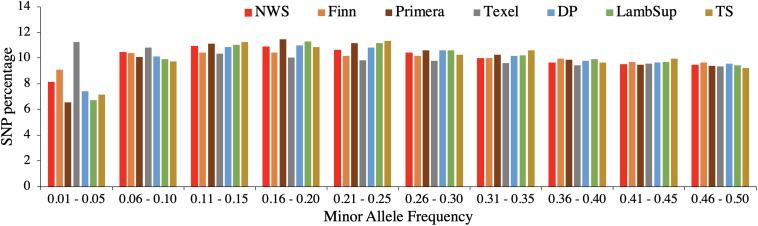
Distribution of single nucleotide polymorphisms (SNP percentage) by minor allele frequency ranges in the Norwegian White Sheep (NWS) and New Zealand sheep populations. Sheep populations from New Zealand are: Finn, Primera, Texel, “Other Dual Purpose” (DP), Lamb Supreme (LambSup), and “Other Terminal Sire” (TS).

The genetic diversity metrics estimated for NWS and NZC sheep populations are summarized in Table 2. The average distances between adjacent SNPs were similar across populations and ranged from 0.023 Mb (NWS, “Other Dual Purpose”, Lamb Supreme, and “Other Terminal Sire”) to 0.025 Mb (Finn). The average LD between adjacent SNPs ranged from 0.156 (Primera) to 0.231 (Finn). Among all NZC populations, “Other Dual Purpose” and “Other Terminal Sire” Composites presented the most similar average LD compared to NWS (∼ 0.17). The lowest consistency of gametic phase was estimated between NWS and Finn (0.397), and between NWS and Texel (0.443), respectively. Similar consistency of gametic phase was estimated between NWS and the other NZC populations (∼ 0.52). The distribution of SNPs by MAF ranges is shown in Figure 1. The proportion of polymorphic SNPs was lower in the Finn and Texel breeds (83.6 and 88.6%, respectively), and similar among the other populations (∼ 94.0%). However, the distribution of SNP percentage was approximately constant by MAF ranges in the different populations (Figure 1).

**TABLE 2:** Average distance between single nucleotide polymorphisms (Dist, in Mb), average linkage disequilibrium (LD), consistency of gametic phase (GP), proportion of polymorphic SNPs (Polim,%), observed (HO) and expected (HE) heterozygosity, average pairwise genetic distance (DST), and inbreeding coefficients estimated based on excess of homozygosity (FE), variance of additive genotypes (FG), and runs of homozygosity (FROH), for Norwegian White Sheep (NWS) and New Zealand Composite sheep populations.

	**NWS**	**Finn**	**Primera**	**Texel**	**DP**	**LambSup**	**TS**
^1^Dist	0.023 (0.016)	0.025 (0.018)	0.024 (0.017)	0.024 (0.017)	0.023 (0.016)	0.023 (0.016)	0.023 (0.016)
^1^LD	0.174 (0.242)	0.231 (0.288)	0.156 (0.228)	0.222 (0.281)	0.172 (0.239)	0.167 (0.235)	0.177 (0.242)
^2^GP	–	0.397	0.548	0.443	0.526	0.538	0.512
Polim	93.50%	83.60%	94.80%	88.60%	94.20%	94.40%	93.50%
HO	0.333 (0.142)	0.346 (0.164)	0.352 (0.141)	0.330 (0.157)	0.333 (0.138)	0.347 (0.14)	0.340 (0.143)
HE	0.335 (0.142)	0.331 (0.148)	0.343 (0.136)	0.325 (0.151)	0.339 (0.14)	0.342 (0.137)	0.340 (0.139)
DST	0.270 (0.011)	0.263 (0.023)	0.271 (0.005)	0.259 (0.018)	0.274 (0.013)	0.272 (0.007)	0.274 (0.018)
FE	0.007 (0.031)	−0.046 (0.035)	−0.018 (0.016)	−0.016 (0.038)	0.017 (0.051)	−0.014 (0.021)	0.001 (0.048)
FG	0.007 (0.103)	−0.042 (0.107)	−0.018 (0.026)	−0.012 (0.184)	0.016 (0.076)	−0.012 (0.032)	0.001 (0.107)
FROH	0.001 (0.000)	0.020 (0.007)	0.000 (0.000)	0.004 (0.001)	0.001 (0.000)	0.000 (0.000)	0.006 (0.004)

*^1^Dist and LD were estimated between adjacent single nucleotide polymorphisms. ^2^GP was estimated in distances lower than 0.02 Mb, between NWS and New Zealand sheep populations. Sheep populations from New Zealand are: Finn, Primera, Texel, “Other Dual Purpose” (DP), Lamb Supreme (LambSup), and “Other Terminal Sire” (TS). Standard deviations in brackets.*

The H_O_ was lower than the H_E_ for NWS and “Other Dual Purpose” (Table 2). All populations had a similar average pairwise genetic distances (∼ 0.27). In general, inbreeding coefficients estimated based on the excess of homozygosity and variance of additive genotype were similar across populations. In addition, populations with H_O_ lower than H_E_ showed negative inbreeding coefficients estimated based on these methods (i.e., Finn, Primera, Texel, and Lamb Supreme). Low inbreeding coefficients were obtained for ROH-based inbreeding. Finn had the highest, and “Other Terminal Sire” and NWS the lowest levels of genomic inbreeding.

#### Detailed Study of NWS Inbreeding Coefficients

Due to the lack of reports on inbreeding levels in NWS, a detailed description will be provided here. The average (SD) pedigree-based inbreeding coefficients for the NWS were 0.009 (0.019) and 0.027 (0.025), considering all and only genotyped animals, respectively (up to 27 generations back). Pearson correlations between estimated inbreeding coefficients using different methods for the NWS are shown in Supplementary Table S1.

As expected, inbreeding coefficients estimated based on the excess of homozygosity and ROH had the highest correlation (0.99; Supplementary Table S1). On the other hand, correlations calculated between inbreeding coefficients estimated based on the variance of additive genotypes and the other methods were negative and of low magnitude (ranging from −0.15 to −0.37). The number of NWS genotyped animals and average inbreeding coefficients per birth year are presented in Supplementary Figure S1.

The majority of NWS genotyped animals were born in 2016 (∼ 35%). In addition, a strong decrease in inbreeding estimated based on the variance of additive genotypes was observed after 1998. The average inbreeding coefficients estimated based on the ROH was almost constant over time (∼ 0.01). A slight increase in pedigree- and excess of homozygosity-based inbreeding was observed over time, but still with a low average of 0.0011 and 0.0008 over years, respectively.

#### LD and Consistency of Gametic Phase

**FIGURE 2:**
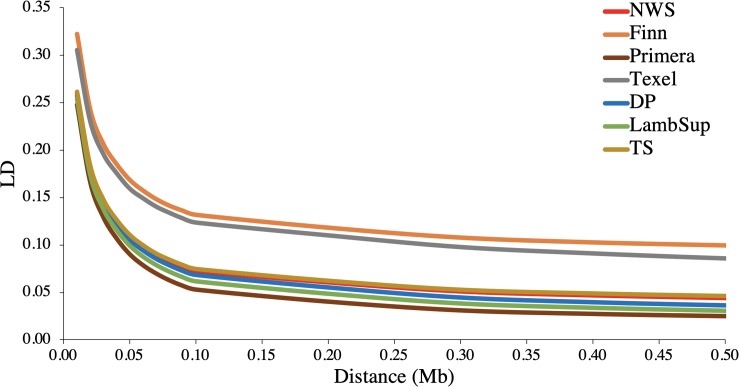
Average linkage disequilibrium (LD) at given distances for Norwegian White Sheep (NWS) and New Zealand sheep populations. Sheep populations from New Zealand are: Finn, Primera, Texel, “Other Dual Purpose” (DP), Lamb Supreme (LambSup), and “Other Terminal Sire” (TS).

The LD decay pattern for all populations is shown in Figure 2. In general, the highest LD was observed for Finn (ranged from 0.322 to 0.100) and Texel (ranged from 0.305 to 0.086). The LD decay pattern for NWS was similar to the observed for “Other Terminal Sire” and “Other Dual Purpose”. Primera had the lowest LD levels across most distances and ranged from 0.248 to 0.025. At the average distance between adjacent SNPs (∼0.02 Mb), the average LD estimates were moderate in all populations (>0.15).

**FIGURE 3:**
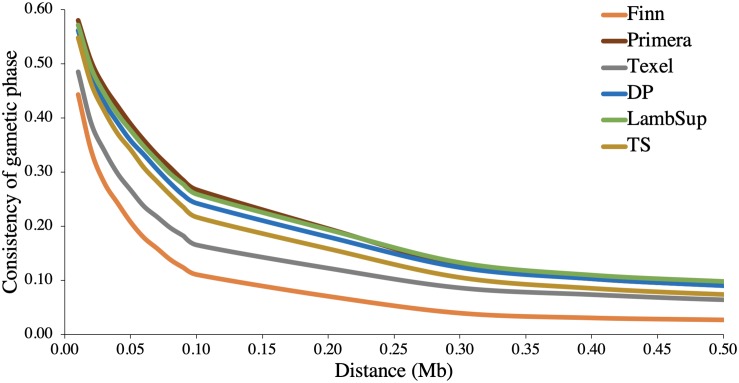
Consistency of gametic phase at given distances between Norwegian White Sheep (NWS) and New Zealand sheep populations. Sheep populations from New Zealand are: Finn, Primera, Texel, “Other Dual Purpose” (DP), Lamb Supreme (LambSup), and “Other Terminal Sire” (TS).

The consistency of gametic phase between NWS and the NZC sheep populations is shown in Figure 3. Among all NZC sheep populations, Finn had the lowest consistency of gametic phase with the NWS at all analyzed distances (ranging from 0.443 to 0.026). On the other hand, Primera, Lamb Supreme, and “Other Dual Purpose” NZC populations had the highest consistency of gametic phase with NWS, respectively (ranging from 0.580 to 0.090, 0.571 to 0.098, and 0.561 to 0.090, respectively).

#### ROH

**FIGURE 4:**
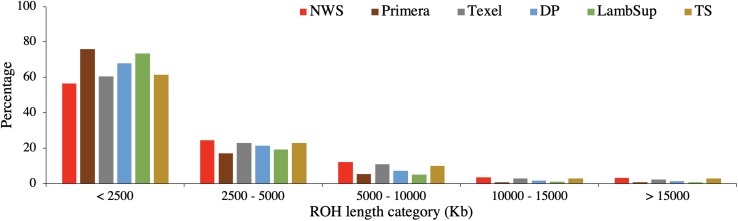
Proportion of runs of homozygosity segments in each length category for the Norwegian White Sheep (NWS) and New Zealand sheep populations. Sheep populations from New Zealand are: Finn, Primera, Texel, “Other Dual Purpose” (DP), Lamb Supreme (LambSup), and “Other Terminal Sire” (TS).

The descriptive analysis of the ROH is summarized in Table 3. The proportion of ROH segments in each length category for NWS and NZC sheep populations are shown in Figure 4. As the number of genotyped animals can influence the ROH detection, 150 randomly selected animals from each population were also used to estimate ROH (Supplementary Table S2 and Supplementary Figure S2).

**TABLE 3:** Descriptive statistics of the runs of homozygosity (ROH) for the Norwegian White Sheep (NWS) and New Zealand sheep populations.

	**NWS**	**Finn**	**Primera**	**Texel**	**DP**	**LambSup**	**TS**
Ntotal	38,188	2,585	139,971	20,071	103,033	197,510	6,939
Min SNPs	50	35	55	46	54	56	41
nSEG	48.2 [0–85]	51.7 [29–78]	16.4 [0–89]	91.2 [32–131]	56.3 [2–132]	32.4 [3–82]	42.1 [8–136]
Kbi	177,692 [0–647,569]	217,651 [71,553–353,140]	37,609 [0–547,796]	313,411 [82,556–647,105]	164,412 [10,949–773,087]	78,717 [5,295–348,486]	144,698 [13,644–775,800]
Kb	3,685 [1,000–82,746]	4,210 [1,001–67,656]	2,298 [1,000–94,879]	3,435 [1,000–75,723]	2,922 [1,000–131,632]	2,428 [1,000–58,583]	3,441 [1,000–44,028]
nSNP	775 [100–17,105]	811 [101–13,401]	443 [100–18,753]	694 [100–15,323]	616 [100–27,778]	502 [100–12,472]	719 [101–9,515]
density	4.9 [3.3–14]	5.4 [3.7–14.6]	5.5 [3.1–16.1]	5.0 [3.3–13.8]	4.8 [3.0–16.8]	5.0 [3.1–21.2]	4.9 [3.3–13.9]
phom	0.997 [0.893–1]	0.997 [0.963–1]	0.996 [0.874–1]	0.997 [0.940–1]	0.997 [0.912–1]	0.997 [0.890–1]	0.997 [0.932–1]

*Sheep populations from New Zealand are: Finn, Primera, Texel, “Other Dual Purpose” (DP), Lamb Supreme (LambSup), and “Other Terminal Sire” (TS). Ntotal: total number of segments. Min SNPs: minimum number of single nucleotide polymorphisms (SNP) in a ROH, calculated as suggested by Lencz et al. (2007). nSEG: average number of segments for the individual declared homozygous. Kbi: average size of total homozygous segments per individual. Kb, average of total number of kb contained within homozygous segments. nSNP, average number of SNPs in run. Density, inverse SNP density in Kb/SNP. Phom, proportion of sites homozygous. Minimum and maximum values are shown inside brackets.*

As expected, a higher number of ROH segments were observed when including all the available genotypes in the analysis. However, the average number and size of segments, the average number of SNPs in a ROH, proportion of sites homozygous and the proportion of ROH segments in each ROH length category were similar in both scenarios (Table 3, Figure 4, and Supplementary Table S2, respectively).

The variation in the number of ROH segments observed in the NWS was similar to the variation observed in Primera and Lamb Supreme. However, the average number of ROH segments was higher for NWS (48.3) than Primera (16.4) and Lamb Supreme (32.4). In general, there was a large variability in the average genome size covered by homozygous segments across populations (Table 3). The maximum genomic region covered by ROH segments were observed in “Other Terminal Sire” (775,800 kb) and “Other Dual Purpose” (773,087 kb) animals, which are from breeds formed by numerous small-sized breeds. The NWS showed moderate average of total length of segments (177,692 kb). However, a high variability was observed among individuals. The average SNP density (number of SNPs per kb) and the proportion of homozygous sites were similar across all populations (∼ 5 SNPs/kb, and ∼0.997, respectively).

The majority of ROH segments observed in the composite breeds had short length (i.e., segments were shorter than 2,500 kb), indicating ancient inbreeding. Primera, Lamb Supreme, and “Other Dual Purpose” had the highest proportion of short segments compared to the other sheep populations, which is likely associated with ancient inbreeding. In all populations, only a small proportion of ROH segments were longer than 10,000 kb. Primera and Lamb Supreme had the lowest proportion of long segments (>2,500 kb; Figure 4).

### Clustering Populations and Admixture Analysis

#### PCA

**FIGURE 5:**
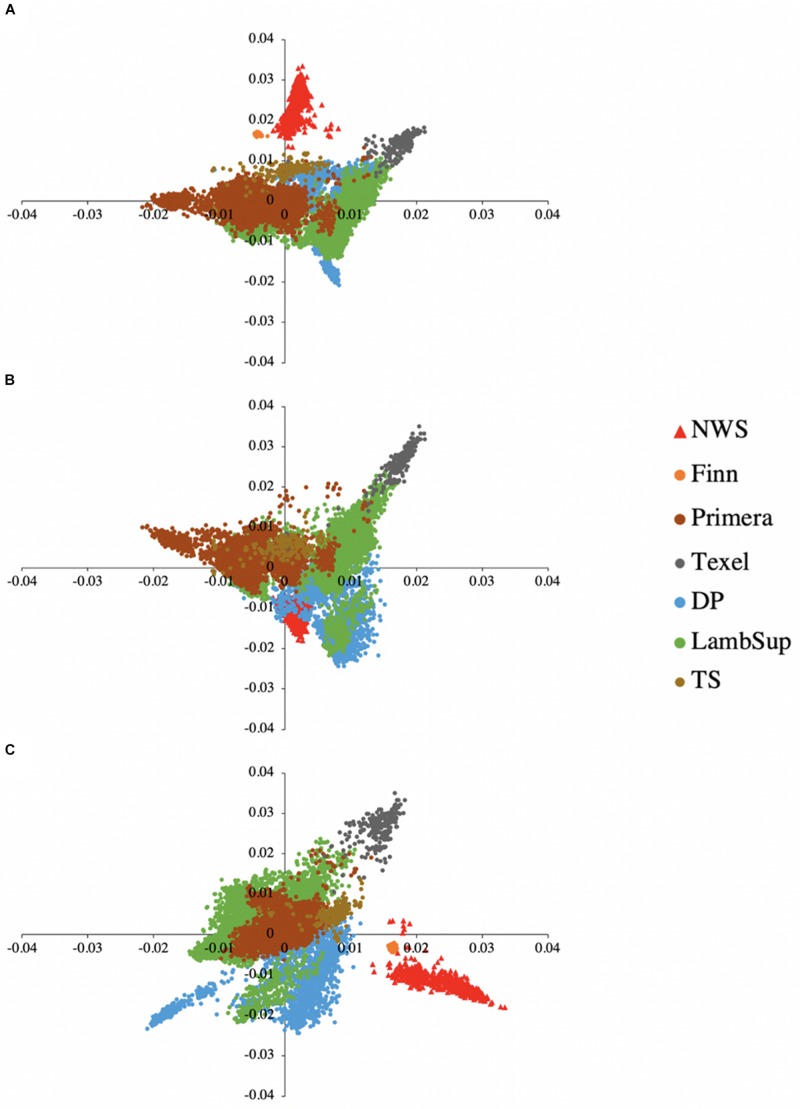
Principal component decomposition of the genomic relationship matrix colored by breed. Breeds from Norway (represented as red triangular dots): Norwegian White Sheep (NWS). New Zealand sheep populations (represented as circular dots): Finn, Primera, Texel, “Other Dual Purpose” (DP), Lamb Supreme (LambSup), and “Other Terminal Sire” (TS). Letters in the figure represent the decomposition of the first and second **(A)**, first and third **(B)**, and second and third **(C)** principal components, respectively.

The principal component decomposition of the genomic relationship matrix into the first three principal components is shown in Figure 5. The first, second and third principal components explained 21.65, 12.68, and 9.32% of the total genomic variance, respectively. In general, the plot of the first and second (Figure 5A), and second and third (Figure 5C) principal components partially discriminate NWS, Finn, and the other NZC populations. However, the first and third principal components (Figure 5B) shows a common clustering among individuals from all populations.

#### Admixture Analysis

**FIGURE 6:**
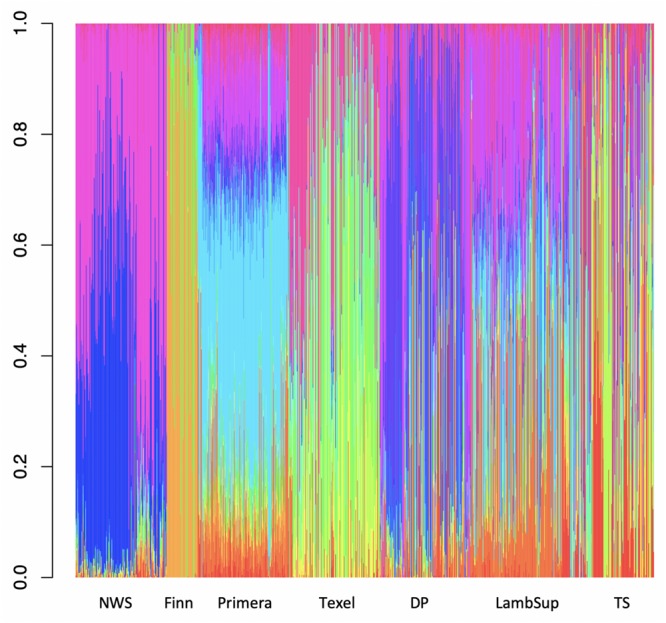
Breed composition per animal calculated for Norwegian White Sheep (NWS) and different New Zealand sheep populations. Sheep populations from New Zealand are: Finn, Primera, Texel, “Other Dual Purpose” (DP), Lamb Supreme (LambSup), and “Other Terminal Sire” (TS).

Among all number of ancestral populations compared (i.e., *k* = 1, 2, …, 25), *k* = 21 had the lowest cross-validation error (Supplementary Figure S3), and therefore, it was used to represent the optimal number of ancestral populations in this study. The individual breed composition based on *k* = 21 is presented in Figure 6. Finn and Texel seem to have originated from a similar genetic resource, based on a lower number of ancestral populations compared to NWS and other NZC sheep populations. The ancestral populations that originated the NWS are similar to the ancestral populations that contribute in the development of “Other Dual Purpose”, Primera and Lamb Supreme.

#### Genomic Population Tree

**FIGURE 7:**
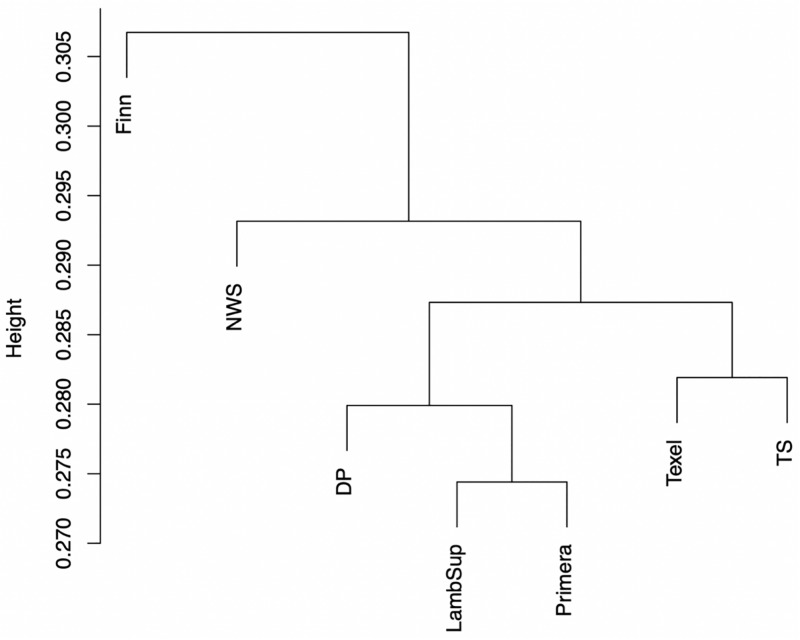
Genomic population tree comparing the genomic distance between Norwegian White Sheep (NWS) and different New Zealand sheep populations. Sheep populations from New Zealand are: Finn, Primera, Texel, “Other Dual Purpose” (DP), Lamb Supreme (LambSup), and “Other Terminal Sire” (TS).

The genomic population tree constructed based on the genomic distance estimated between NWS and the different NZC sheep populations is presented in Figure 7. In summary, the Lamb Supreme was grouped close to Primera, while Texel was grouped close to “Other Terminal Sire” breed group. The Lamb Supreme and Primera composite breeds were closer to “Other Dual Purpose” than Texel and “Other Terminal Sire” breed groups. In addition, Figure 7 shows a greater differentiation between Finn and NWS and the other NZC sheep populations. In this context, the NWS breed seems to be more related to the NZC populations than Finn.

### Signatures of Selection

#### F_ST_ Statistic

**FIGURE 8:**
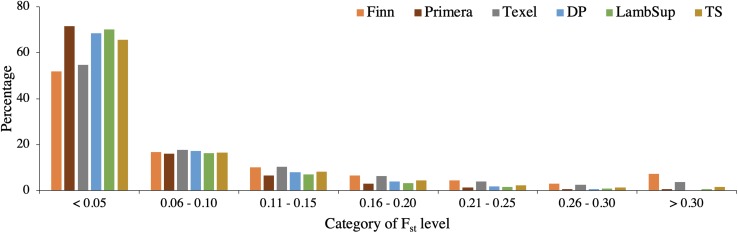
Distribution of F_ST_ values for the Norwegian White Sheep (NWS) and New Zealand sheep populations. Sheep populations from New Zealand are: Finn, Primera, Texel, “Other Dual Purpose” (DP), Lamb Supreme (LambSup), and “Other Terminal Sire” (TS).

A summary of the F_ST_ statistics is shown in Table 4 and the percentage of SNPs falling into each F_ST_ category is illustrated in Figure 8. Most SNPs had very low F_ST_ level (<0.10; Figure 8), indicating that only a few genomic regions were potentially fixed due to intensive selection pressure. The majority of genomic regions were identified when contrasting NWS and Finn (5.19%), and NWS and Texel (2.20%). However, it is important to point out that the average of the F_ST_ statistics considering only the selected SNPs was low, even for those breeds (0.47 for NWS and Finn, and 0.41 for NWS and Texel).

**TABLE 4:** Mean and standard deviation (inside brackets) of the F_ST_ statistics considering all (F_ST__All_) and only the selected (F_ST__Selected_) single nucleotide polymorphisms (SNP) for the contrasted sheep populations.

**^1^Contrasted populations**	**F_ST__All_**	**Selected (%)**	**F_ST__Selected_**
NWS vs. Finn	0.09 (0.123)	5.19	0.47 (0.107)
NWS vs. Primera	0.04 (0.058)	0.29	0.39 (0.034)
NWS vs. Texel	0.08 (0.092)	2.20	0.41 (0.048)
NWS vs. DP	0.04 (0.055)	0.01	0.36 (0.014)
NWS vs. LambSup	0.04 (0.059)	0.21	0.38 (0.028)
NWS vs. TS	0.05 (0.072)	0.83	0.41 (0.052)

*^1^Norwegian White Sheep (NWS) was contrasted with different New Zealand sheep populations. Sheep populations from New Zealand are: Finn, Primera, Texel, “Other Dual Purpose” (DP), Lamb Supreme (LambSup), and “Other Terminal Sire” (TS). SNP with *F*_**ST**_ values greater than the average plus three standard deviations were selected.*

## Discussion

### Population Characterization and Genetic Diversity Metrics

The average distance between SNPs was similar across populations as all individuals were genotyped using an HD SNP chip panel. Finn presented the greatest average distance between SNPs (0.025 Mb; Table 2), which is a consequence of the larger number of SNPs excluded due to low MAF (Table 1). The larger number of SNPs excluded due to MAF is likely related to the smaller number of genotyped animals and the reduced genetic diversity compared to the other populations. In general, the distribution of SNP percentage was approximately constant by MAF ranges (Figure 1), and the proportion of polymorphic SNPs was high in all analyzed populations (Table 2). Thus, even though the proportion of monomorphic SNPs can be underestimated because not all breeds were included in the development of the HD SNP chip, there is an indication of high genetic diversity in all populations evaluated in this study.

Heterozygosity measures the level of genetic variation within a population. Thus, usually populations developed based on a large number of ancestral populations or under intensive crossbreeding schemes have high H_O_ and H_E_ (Brito et al., 2017a). In this context, the levels of H_O_ and H_E_ were high (>0.32; Table 2). The H_O_ was slightly lower than H_E_ in NWS and “Other Dual Purpose”. Brito et al. (2017a), studying the genetic diversity among Primera, Lamb Supreme, Texel, and “Other Dual Purpose” also reported similar levels of H_O_ and H_E_. Similarly, Prieur et al. (2017), working with Romney, Coopworth, Perendale, and Texel NZC sheep populations, reported levels of heterozygosity around 0.36. Kijas et al. (2012), performing a genome-wide scan for the signatures of selection using 74 diverse breeds from all over the world, reported an average (SD) H_O_ of 0.33 (0.03). Thus, even though the authors did not include the NWS and NZC populations (those studied here), H_O_ estimates found in this study seems to corroborate with their report. On the other hand, higher heterozygosity estimates were reported by Vahidi et al. (2016) (∼ 0.72) and Neubauer et al. (2015) (∼ 0.75), using microsatellites to study Iranian indigenous and Hungarian sheep, respectively.

Usually average pairwise genetic distances have been used to access the genetic distance among populations (e.g., Tolone et al., 2012; Neubauer et al., 2015). However, within-population genetic distance is another metric of genetic diversity. In this study, similar average pairwise genetic distances were estimated in all populations (Table 2), suggesting similar levels of genetic diversity within each population. Gaouar et al. (2016), using microsatellite markers to estimate the population structure and genetic diversity of five Moroccan sheep breeds, reported higher levels of genetic diversity among animals (∼ 0.75). In dairy goats, Brito et al. (2017b) reported similar average pairwise genetic distances to those found in this study, for the Nubian and Toggenburg breeds (∼ 0.26).

#### Inbreeding

Inbreeding can be defined as the probability of an individual receiving, at a given locus, the same ancestral-allele from both parents (Wright, 1922). Several studies have reported the negative effects of inbreeding in sheep (e.g., Drobik and Martyniuk, 2016; Gholizadeh and Ghafouri-Kesbi, 2016), goats (Deroide et al., 2016; Mahmoudi et al., 2018), and cattle (Smith et al., 2010; Pereira et al., 2016; Reverter et al., 2017). Therefore, monitoring inbreeding is important to avoid inbreeding depression. On average, genotyped animals in this study had a low level of genomic inbreeding (Table 2). This might be attributed to the high gene flow between different flocks and recent use of crossbreeding in the development of composite populations.

Similar average inbreeding coefficients were estimated based on the excess of homozygosity and variance of additive genotype, which may be related to the fact that both approaches use the same SNP information (Purcell et al., 2007; VanRaden, 2008). The inbreeding based on ROH is highly dependent on the ROH length, which can change with the population (Rodríguez-Ramilo et al., 2019). Finn had the highest level of genomic inbreeding based on all metrics. This could be due to the reduced sample size and sampling approach (e.g., few flocks sampled). Nonetheless, careful mating decisions are advised especially in this breed.

Differences in the pattern of inbreeding coefficients over the years (Supplementary Figure S1) highlight the need of using different methods to better understand the levels of inbreeding in the flock. It is important to note that founder animals were assumed unrelated in this study, which explains the pedigree inbreeding values of zero in the first years. In this context, using different methods to deal with founder animals, such as meta-founders (Legarra et al., 2015; Van Grevenhof et al., 2019), might more accurately model the inbreeding level based on pedigree information. ROH-based inbreeding was similar over time, which might be due to the low levels of inbreeding in these populations.

#### LD and Consistency of Gametic Phase

The accuracy of genomic predictions and the power of QTL detection in genome-wide association studies are partially determined by the levels of LD in a population (Goddard, 2009). Usually crossbreed populations exhibit faster LD decay compared to pure breeds (Prieur et al., 2017). The largest LD values observed for Finn and Texel (Table 2 and Figure 2) indicate less independent segregation between SNP markers and QTLs. The low to moderate LD levels indicate that large training populations might be required to obtain accurate genomic breeding values (Meuwissen et al., 2001; VanRaden et al., 2009).

The performance of across-population genomic predictions are highly dependent not only on the levels of LD, but also on the consistency of gametic phase. The consistency of gametic phase measures the association between SNPs and QTLs alleles across breeds, as well as the QTL effects between breeds (Brito et al., 2017a). Thus, if the genetic distance between populations is large, the linkage phase will not be consistent across populations over long distances in the genome. The low consistency of gametic phase estimated between NWS and Finn indicates that there might be no improvement in the performance of genomic predictions by combining both breeds in a single training population. The consistency of gametic phase estimated among NWS and the other NZC populations was moderate (Table 2 and Figure 3), indicating a potential benefit on using a common training population for genomic predictions. This is even more important for smaller training populations (reduced number of animals with genotypes and phenotypes for certain traits and populations) and has yielded positive results (Lund et al., 2010; Zhou et al., 2019). Furthermore, Kizilkaya et al. (2010) and Toosi et al. (2010) showed, based on simulation studies, that denser SNP panels are needed to perform across-breed genomic predictions, in order to establish a high consistency of gametic phase among SNPs and QTLs in the different breeds.

Similar LD estimates, but higher consistency of gametic phase were found by Brito et al. (2017a) when studying the relatedness between NZC populations. Prieur et al. (2017) reported lower LD estimates (∼ 0.10) in Coopworth, Romney, Perendale, and Texel. The variation in LD and consistency of gametic phase estimates corroborates with Kijas et al. (2012), who found large differences in the estimates among 74 worldwide sheep breeds. No reports were found in the literature for NWS. Sheep LD estimates reported in the literature are usually lower than estimates reported for other livestock species (e.g., Khatkar et al., 2008; Porto-Neto et al., 2013a, b). This might be due to a smaller bottleneck in the domestication process, use of a larger number of breeds and reduced use of reproductive technologies (e.g., artificial insemination).

#### ROH

The ROH pattern contributes to a better understanding of population history (Purfield et al., 2012, 2017; Bjelland et al., 2013). ROH can arise when the same chromosomal segment, inherited from the same common ancestor by both parents, is passed together to the offspring (Broman and Weber, 2002; Rodríguez-Ramilo et al., 2019). Short ROH are usually related to ancient inbreeding as the probability of recombination from repeated meiosis events will “break-up” the chromosomal segments (Purfield et al., 2012; Rodríguez-Ramilo et al., 2019). On the other hand, long ROH segments are related to recent inbreeding. Longer average ROH segments were observed for Texel and Finn (Table 3). However, this might be due to the reduced sample size and sampling process (previously mentioned). The highest proportions of short ROH segments observed for Primera, Lamb Supreme, and “Other Dual Purpose” (Figure 4) indicate that these populations are not highly affected by recent inbreeding.

The similar ROH results observed when using all genotyped animals or a random sample (Tables 3, Supplementary Table S2, Figures 4, and Supplementary Figure S2) indicates that the latter can be used to accurately estimate ROH, in order to speed up the analysis. However, the number of ROH segments identified per animal in each population (Table 3) is related to the number of animals used in the analysis. Comparing ROH results from different studies is challenging as there are multiple factors that can affect the identification of ROH, including the genotype quality control (Albrechtsen et al., 2010), the number of heterozygous genotypes (Purfield et al., 2012), and the different thresholds imposed during the sequence analysis (Howrigan et al., 2011). Therefore, as suggested by Rodríguez-Ramilo et al. (2019), there is a great need to establish consistent criteria to identify and quantify ROH. The criteria used in this study were similar to those used by Brito et al. (2017b).

### Clustering Populations and Admixture Analysi*s*

#### PCA

A partial discrimination between NWS, Finn, and the other NZC populations was observed when analyzing the first and second, and second and third principal components (Figure 5). However, the first and third principal components showed an overlap among individuals from all different sheep populations. These findings suggest that there is moderate genetic similarity between these populations, which may be due to their reasonably similar development history.

#### Admixture Analysis

The choice of the optimal number of ancestral populations is a notoriously difficult statistical problem, which also requires knowledge on the populations’ history (Pritchard et al., 2000; Alexander et al., 2009; Brito et al., 2015). The large optimal number of ancestral populations (*k* = 21) is likely due to the fact that the populations studied here are, mainly composite breeds formed based on multiple (>20; Brito et al., 2017a) founder breeds with different origins. Finn and Texel seemed to have a lower number of ancestral populations, which may be because these are the most specialized breeds included in this study. This is supported by the historical process reported by Brito et al. (2017a) and Prieur et al. (2017) for the NZC Texel breed. The ancestral populations from NWS and “Other Dual Purpose” were similar, especially for the great amount of contribution from the ancestral populations represented by the blue and pink colors in Figure 6. In this context, Brito et al. (2017a), studying the history of NZC breeds, reported that the most common breeds that contributed to the “Other Dual Purpose” population were Coopworth, Romney, Highlander and Landmark.

#### Genomic Population Trees and Signatures of Selection

The genomic population tree constructed based on the genomic distance estimated between NWS and the different NZC sheep populations showed that there is some differentiation between Finn and NWS and the other NZC sheep populations (Figure 7). In this context, NWS appears to be more related to the other NZC populations than to Finn. Most SNPs had very low F_ST_ values (Figure 8), and the average of F_ST_ statistics considering only the selected SNPs was low (Table 4). These findings suggest that no genomic regions were potentially under selection in the studied populations.

## Conclusion

Relatively high genetic diversity was observed within each sheep population. The NWS breed seems to be moderately related to the NZC sheep populations, especially Primera, Lamb Supreme and “Other Dual Purpose”. The moderate genetic relationship between populations from both countries is likely due to the high number of ancestral breeds used in their development. The findings reported here indicate a promising opportunity for collaborative genomic analyses involving NWS and NZC sheep populations.

## Data Availability Statement

The data supporting the results of this article are included within the article and in its Supplementary Material. The raw data cannot be made available, as it is property of the sheep producers in New Zealand and Norway and this information is commercially sensitive.

## Author Contributions

HO, JM, JJ, TB, and LB conceived and designed this study. HO carried out the analyses. HO and LB wrote the manuscript. HO, JM, JJ, TB, TM, NP, SC, and LB interpreted and discussed the results. All the authors reviewed and approved the final manuscript.

## Conflict of Interest

JM and SC were employed by the company AgResearch and NP was employed by the company Focus Genetics. The remaining authors declare that the research was conducted in the absence of any commercial or financial relationships that could be construed as a potential conflict of interest.
