# Supplementary figures and images for: Genetic Connectedness Between Norwegian White Sheep and New Zealand Composite Sheep Populations With Similar Development History
Source: Front Genet. 2020 Apr 24;11:371. doi: 10.3389/fgene.2020.00371 (PMC7194024; doi:10.3389/fgene.2020.00371)

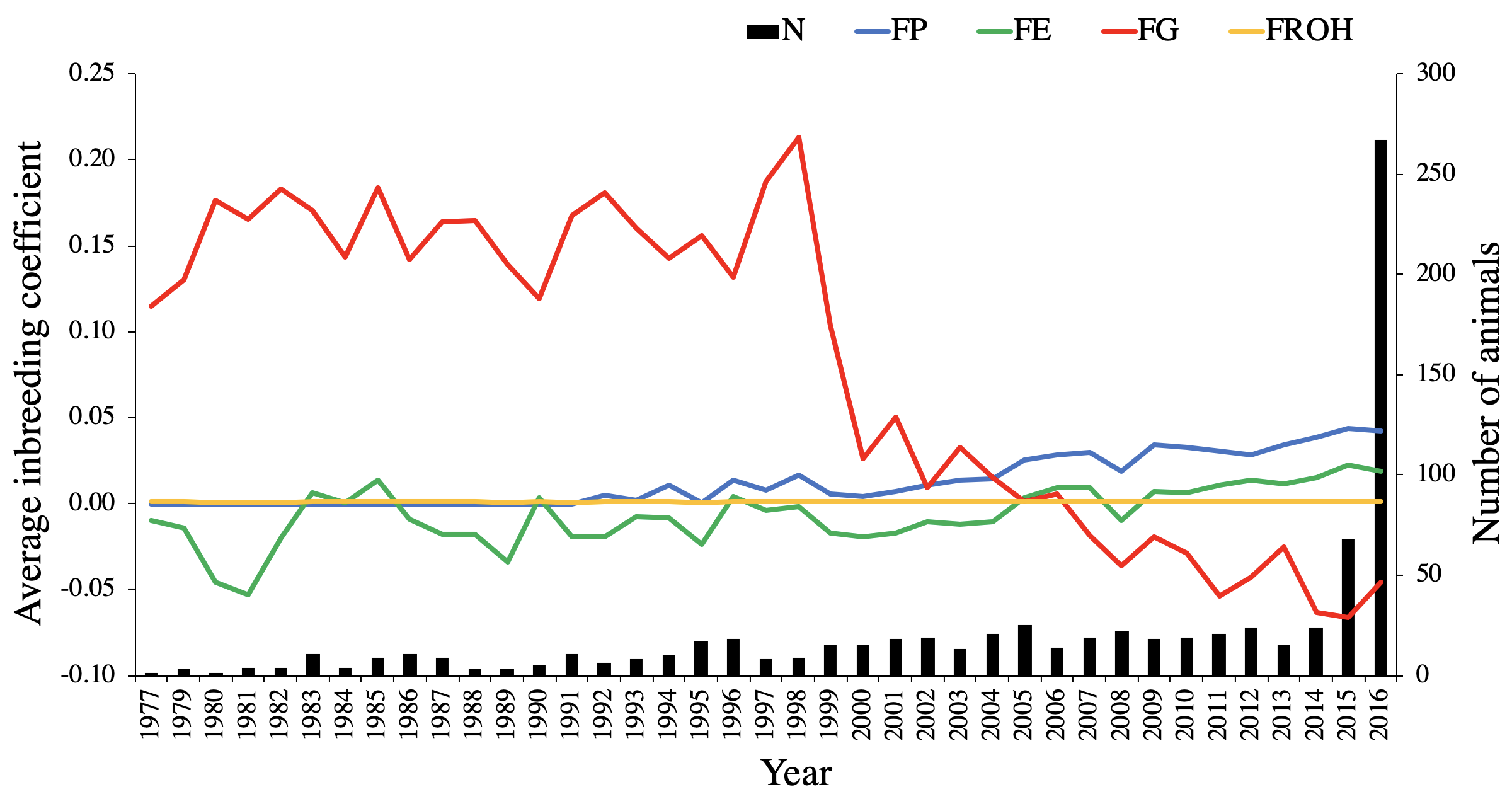

Supplement: Supplementary file 1 [file Image_1.JPEG]

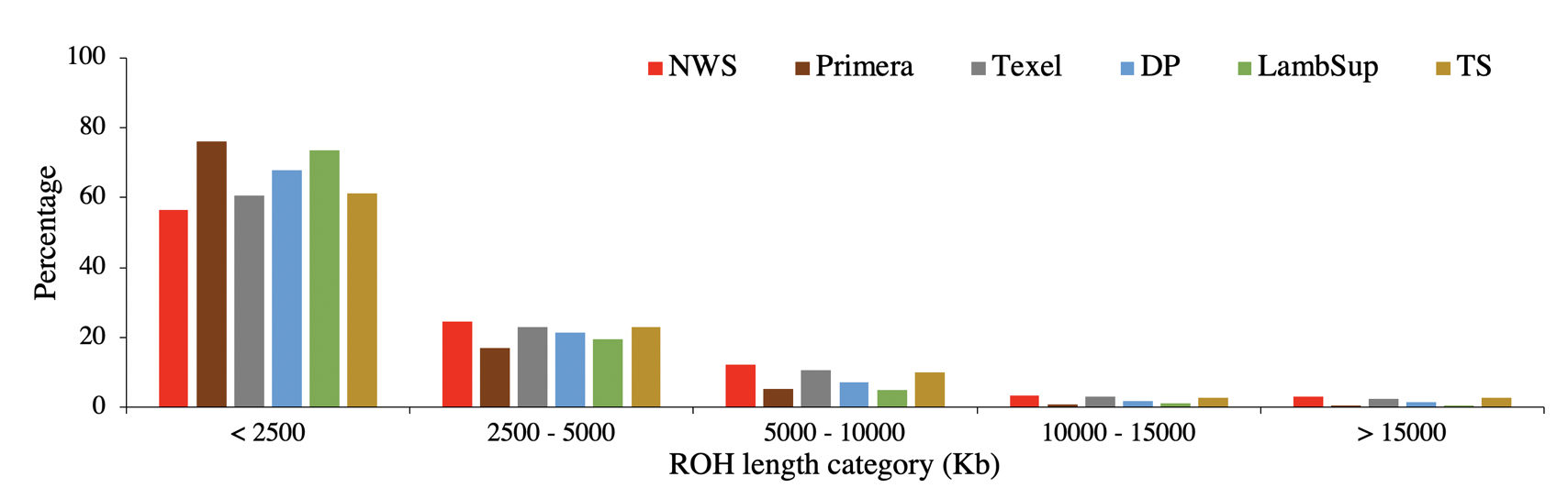

Supplement: Supplementary file 2 [file Image_2.JPEG]

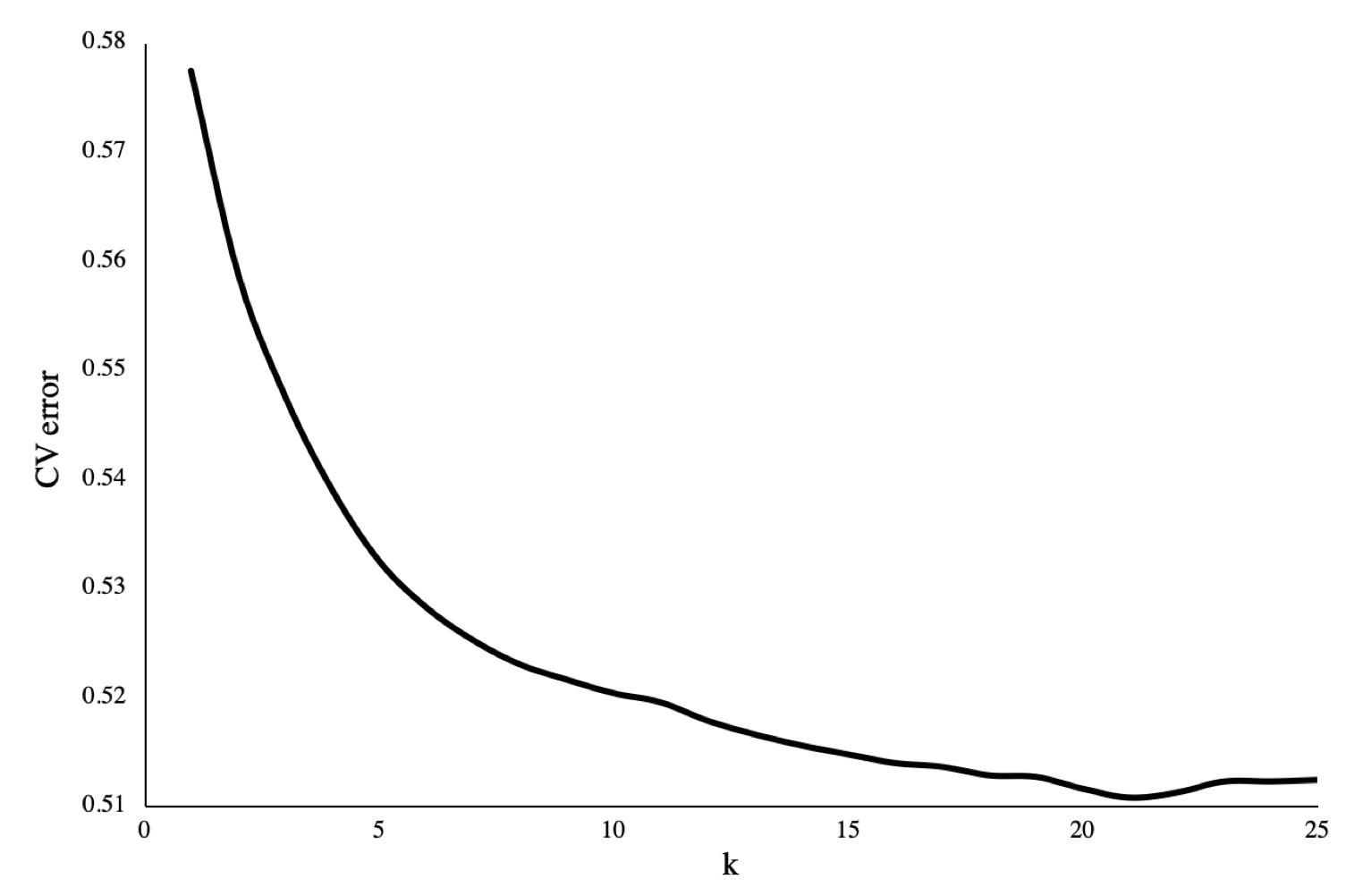

Supplement: Supplementary file 3 [file Image_3.JPEG]
