# Supplementary material for: Genetic Connectedness Between Norwegian White Sheep and New Zealand Composite Sheep Populations With Similar Development History
Source: Front Genet. 2020 Apr 24;11:371. doi: 10.3389/fgene.2020.00371 (PMC7194024; doi:10.3389/fgene.2020.00371)
Supplement: Supplementary file 4 [file Table_1.docx]

**Table S1.** Pearson correlations between inbreeding coefficients estimated based on different methods for the Norwegian White Sheep.

|  | **FP** | **FE** | **FG** | **FROH** |
| --- | --- | --- | --- | --- |
| **FP** | 1.00 |  |  |  |
| **FE** | 0.65 | 1.00 |  |  |
| **FG** | -0.37 | -0.16 | 1.00 |  |
| **FROH** | 0.66 | 0.99 | -0.15 | 1.00 |

Inbreeding coefficients were estimated based on pedigree (FP), excess of homozygosity (FE), variance of additive genotypes (FG), and runs of homozygosity (FROH).
