# Supplementary material for: Genetic Connectedness Between Norwegian White Sheep and New Zealand Composite Sheep Populations With Similar Development History
Source: Front Genet. 2020 Apr 24;11:371. doi: 10.3389/fgene.2020.00371 (PMC7194024; doi:10.3389/fgene.2020.00371)
Supplement: Supplementary file 5 [file Table_2.docx]

**Table S2.** Descriptive analysis of the runs of homozygosity (ROH) for the Norwegian White Sheep (NWS) and New Zealand sheep populations, considering only 150 genotyped animals from each population.

|  | **NWS** | **Primera** | **Texel** | **DP** | **LambSup** | **TS** |
| --- | --- | --- | --- | --- | --- | --- |
| **Ntotal** | 7,403 | 2,518 | 13,975 | 7,943 | 4,901 | 6,159 |
| **Min SNPs** | 42 | 37 | 44 | 42 | 39 | 40 |
| **nSEG** | 49.4 [19 -85] | 16.8 [0 -61] | 93.2 [40 -131] | 53 [9 -128] | 32.7 [6 -74] | 41.1 [8 -136] |
| **Kbi** | 182,953 [37,003 - 626,607] | 38,861 [0 - 242,295] | 317,727 [101,739 -647,014] | 150,363 [13,767 -567,441] | 79,387 [8,266 -295,677] | 139,281 [13,644 -684,296] |
| **Kb** | 3,707 [1,000 – 71,976] | 2,315 [1,000 - 33,882] | 3,410 [1,000 – 49,852] | 2,839.5 [1,000 -58,542] | 2429 [1,000 -43,985] | 3,392 [1,000 - 43,522] |
| **nSNP** | 783 [101 -15,582] | 482 [100 -7,241] | 691 [103 -10,553] | 600 [101 -12,063] | 513 [100 -9,272] | 711 [101 -9,099] |
| **density** | 4.8 [3.3 -13.8] | 5.1 [3.4 -13.4] | 4.9 [3.2 -13.2] | 4.8 [3.2 -13.8] | 4.9 [3.3 -13.4] | 4.9 [3.3 -13.9] |
| **phom** | 0.997 [0.917-1] | 0.996 [0.933-1] | 0.997 [0.939-1] | 0.996 [0.963-1] | 0.996 [0.921-1] | 0.997 [0.932-1] |

Sheep populations from New Zealand are: Finn, Primera, Texel, “Other Dual Purpose” (DP), Lamb Supreme (LambSup), and “Other Terminal Sire” (TS). Ntotal: total number of segments. Min SNPs: minimum number of single nucleotide polymorphisms (SNP) in a ROH, calculated as suggested by Lencz et al. (2007). nSEG: average number of segments for the individual declared homozygous. Kbi: average size of total homozygous segments per individual. Kb: average of total number of kb contained within homozygous segments. nSNP: average number of SNPs in run. Density: inverse SNP density in Kb/SNP. Phom: proportion of sites homozygous. Minimum and maximum values are shown inside brackets.
